# Use of an intraoperative navigation system for retrieving a broken dental instrument in the mandible: a case report

**DOI:** 10.1186/s13256-016-1182-2

**Published:** 2017-01-15

**Authors:** Shintaro Sukegawa, Takahiro Kanno, Akane Shibata, Kenichi Matsumoto, Yuka Sukegawa-Takahashi, Kyosuke Sakaida, Yoshihiko Furuki

**Affiliations:** 1 Division of Oral and Maxillofacial Surgery, Kagawa Prefectural Central Hospital, 1-2-1, Asahi-machi, Takamatsu, Kagawa 760-8557, Japan; 2 Department of Oral and Maxillofacial Surgery, Shimane University Faculty of Medicine, Shimane, Japan

**Keywords:** Surgical navigation system, Broken dental instrument, Foreign body retrieval, Case report

## Abstract

**Background:**

A fracture of root canal instruments, with a fractured piece protruding beyond the apex, is a troublesome incident during an endodontic treatment. Locating and retrieving them represents a challenge to maxillofacial surgeons because it is difficult to access due to the proximity between the foreign body and vital structures. Although safe and accurate for surgery, radiographs and electromagnetic devices do not provide a precise three-dimensional position. In contrast, computer-aided navigation provides a correlation between preoperatively collected data and intraoperatively encountered anatomy.

However, using a navigation system for mandible treatment is difficult as the mobile nature of the mandible complicates its synchronization with the preoperative imaging data during surgery.

**Case presentation:**

This report describes a case of a dental instrument breakage in the mandible during an endodontic treatment for a restorative dental procedure in a 65-year-old Japanese woman. The broken dental instrument was removed using a minimally invasive approach with a surgical navigation system and an interocclusal splint for a stable, identically repeatable positioning of the mandible. Using the three-dimensional position of the navigation probe, a location that best approximated the most anterior extent of the fragment was selected. A minimally invasive vestibular incision was made at this location, a subperiosteal reflection was performed, and the foreign body location was confirmed using a careful navigation system. The instrument was carefully visualized and extruded from the apical to the tooth crown side and was then removed using mosquito forceps through the medullary cavity of the crown side of the tooth. Follow-up was uneventful; her clinical course was good.

**Conclusions:**

The use of a surgical navigation system together with an interocclusal splint enabled the retrieval of a broken dental instrument in a safe and minimally invasive manner without damaging the surrounding vital structures.

## Background

Root canal is one of the most basic dental treatments and is fundamentally dependent on root canal preparation. The treatment comprises the cleaning and shaping of canals. A fracture of root canal instruments is one of the most troublesome incidents during an endodontic treatment. The prevalence of broken instruments ranges from 0.5 to 5% [1–4]. If the broken instrument impedes adequate cleaning of the canal beyond the obstruction, prognosis might be affected by the success of the endodontic treatment [5, 6]. Therefore, some reports have advocated for the retention of broken instruments and fragments. Moreover, in the event that the foreign body migrates into the jawbone beyond the tooth apex, its active removal should be considered to avoid infection and damage to local, vital structures. In the case of a root canal treatment, fractured pieces of instruments protruding beyond the apex are among the most troublesome and frustrating issues that are encountered. Locating and retrieving small foreign bodies (for example, fragments of dental instruments) represents a challenge to maxillofacial surgeons because it is difficult to access and there is a close anatomic relationship between the foreign body and vital structures [7, 8].

For a safe and accurate surgery, the intraoperative use of radiographs and electromagnetic devices may be helpful for removing foreign objects but do not provide a precise three-dimensional position [9, 10]. In contrast, the use of a computer-aided surgical navigation system can provide a correlation between preoperatively collected data and the intraoperatively encountered anatomy.

Here, we report a case of an efficient, minimally invasive retrieval of a broken dental instrument using an intraoperative three-dimensional navigation system and a custom-made interocclusal splint for the reproducibility of the mandibular position.

## Case presentation

**Fig. 1:**
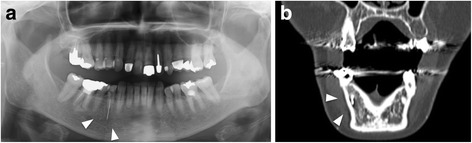
**a** Panoramic radiographs revealed a well-defined fractured instrument lying in the mandible beyond the apical foramen of the right mandibular premolar tooth. **b** The computed tomography image revealed that the fractured segment was located in the mandibular bone

A 65-year-old Japanese woman was referred by her local dentist to our hospital for retrieval of a fragment of Peeso reamer that had broken during a root canal enlargement and that was embedded in the canal of her right mandibular first premolar, extending beyond the apex (tooth number 44). As it was not possible to remove the mandibular foreign body from the root canal at the referral dentist, she was admitted to get it removed using a surgical approach. During the intraoral clinical examination, there was an access cavity filled with a temporary filling material and the tooth was sensitive to pressure. There was tenderness in the apical portion of the tooth. A panoramic radiograph obtained at the initial visit after the patient referral revealed a well-defined fractured instrument lying in the mandible beyond the apical foramen of her right mandibular premolar tooth. Moreover, computed tomography (CT) images revealed that the fractured segment was located within the mandibular bone (Fig. 1). A diagnosis of migration of a broken endodontic instrument beyond the apical foramen into the mandible was made, and surgery was considered necessary to eliminate the symptoms. The decision was made to proceed with the surgery because of increasing pain.

### Treatment

**Fig. 2:**
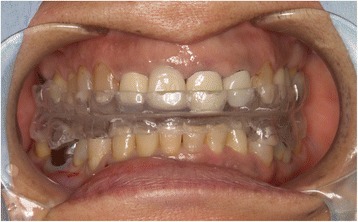
Intraoperative view of the customized interocclusal splint in place

Surgical intervention was required to find the apical root cutting-edge lesion and the broken dental instrument through a small bony window and to remove it from the oral cavity by pushing up in order to reliably preserve the tooth for restorative treatment. Therefore, we decided to use three-dimensional navigation-guided surgery for its minimally invasive nature and high surgical accuracy. As it is difficult to synchronize with the preoperative imaging data during surgery due to the mobile nature of the mandible, we used a customized interocclusal splint for repeatable mandibular positioning while enabling surgical access. As part of the CT imaging preparation for navigation, a customized interocclusal splint was fabricated by pressing acrylic resin into a dental mold at the first visit. First, the created bimaxillary upper and lower jaw splints were adhered using resin at a stable position of the mandible, that is, the position in which the mandible is slightly opened from the central occlusion. A maxillofacial CT was obtained using the customized interocclusal splint to maintain the mandible in a repeatable position, which would be vital for the accuracy required for retrieving the small foreign body (Fig. 2).

**Fig. 3:**
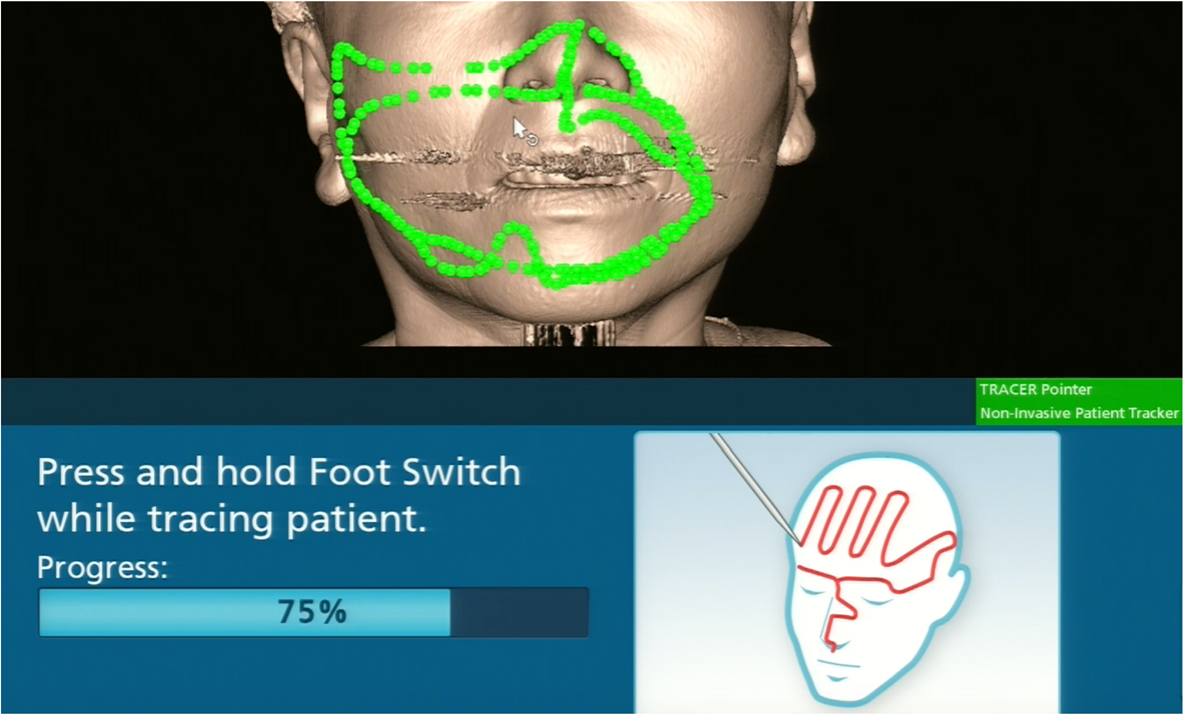
Patient registration with the tracer probe. The *green points* show the trace marked by the probe

The imaging data were obtained in a Digital Imaging and Communication in Medicine format and transferred to a Medtronic StealthStation S7 workstation with Synergy Fusion Cranial 2.2.6 software (Medtronic Navigation Inc., Louisville, CO, United States). Our patient was taken into the operating room where her customized interocclusal splint was reinserted. A Patient Tracker EM was affixed to her forehead to act as a reference array to track the navigation probe. To perform patient-to-CT data registration, the instrumentation navigation probe was used to trace the reference array, soft tissue landmarks of the face, and hard tissue points (for example, tooth cusps and incisal edges) (Fig. 3). After data registration was complete, continuous three-dimensional tracking of the navigation probe was available to the surgeon in real time. This was possible because of the identical position of the mandible during the CT scan and in the operating room due to the use of the interocclusal splint. In this case, the splint was not sterilized but chemically disinfected with benzalkonium chloride as it did not directly contact the surgical site.

**Fig. 4:**
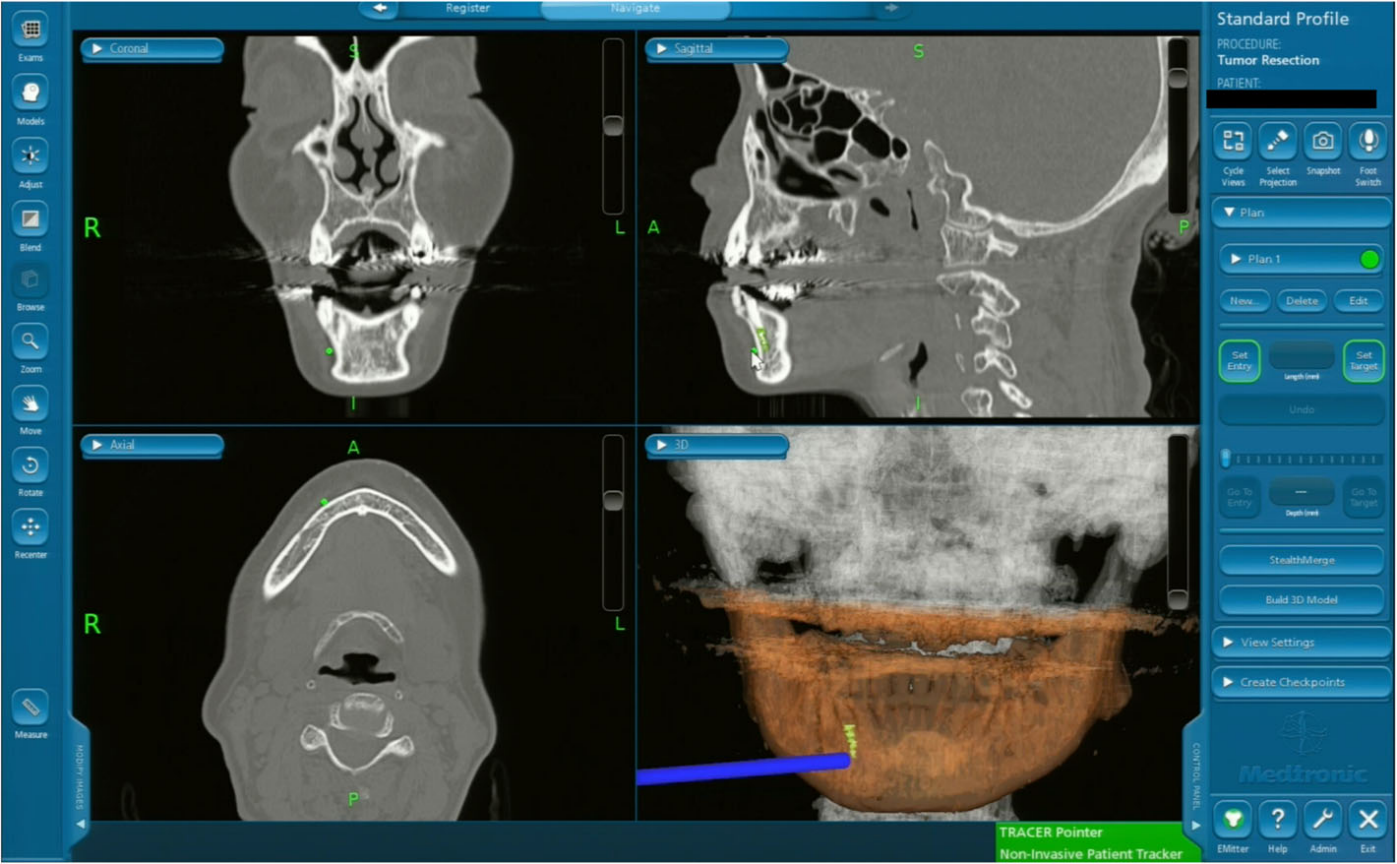
Intraoperative navigation system screenshot showing the multiplane view of the position of the surgeon’s navigation probe (*blue*) in relation to the broken instrument fragment at the time of location

**Fig. 5:**
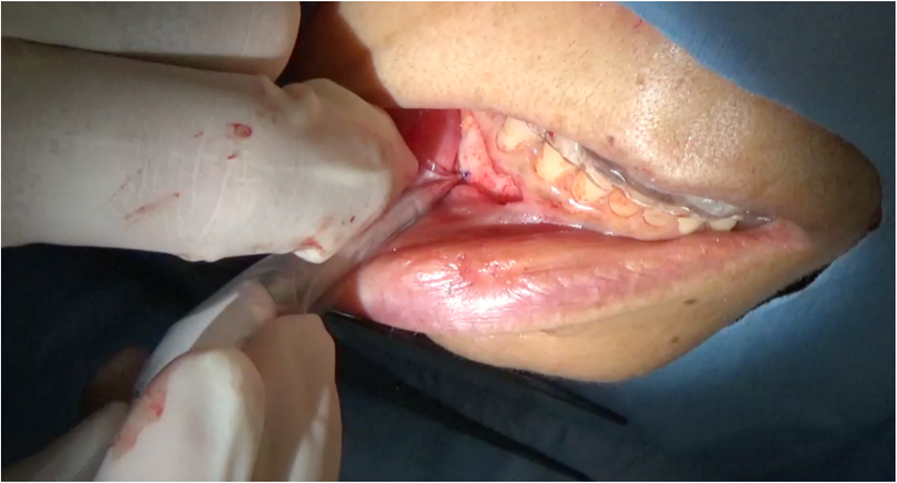
The location of the broken instrument was confirmed using a careful navigation system

**Fig. 6:**
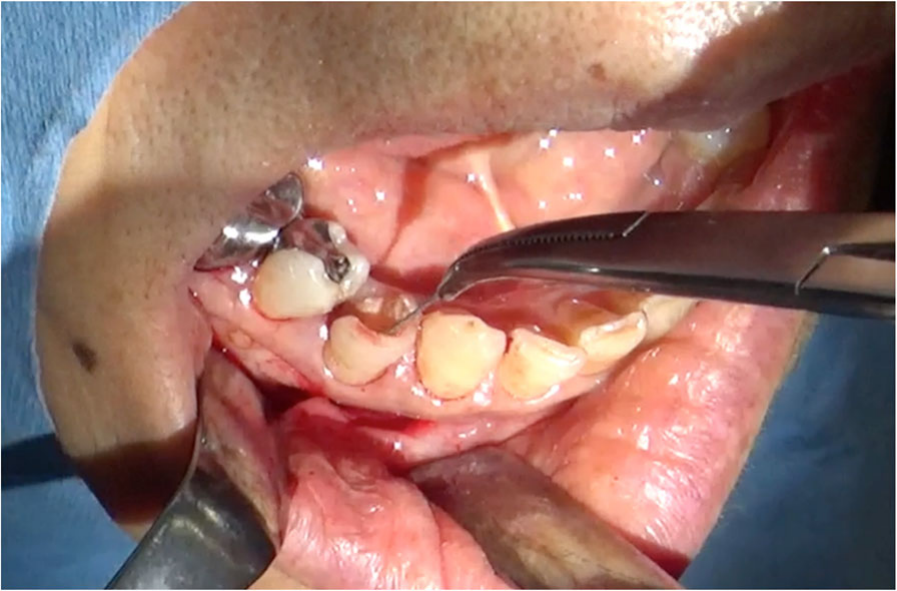
The broken instrument was carefully visualized and extruded from the apical to the crown side of the tooth, then removed with mosquito forceps through the medullary cavity of the crown side of the tooth

Using the three-dimensional location of the navigation probe with respect to the broken instrument fragment, a location was selected that best approximated the most anterior extent of the fragment in conjunction with the navigation probe (Fig. 4). An approximately 15-mm vestibular incision was made in this location, subperiosteal reflection was performed, and the foreign body location was confirmed using a careful navigation system (Fig. 5). A 3-mm bony window was prepared through the buccal cortex that corresponded to the root apex of the right premolar. The instrument was carefully visualized and extruded from the apical to the tooth crown side and was then removed using mosquito forceps through the medullary cavity of the crown side of the tooth (Fig. 6). A postoperative radiograph was obtained to confirm the complete removal of the fractured segment. Our patient was discharged later that day. Follow-up after the prosthetic treatment was uneventful; her clinical course was good.

## Discussion

Endodontic files, burs, and occasionally, other dental instruments tend to break off during surgical procedures because of defective manufacturing, stress, fatigue, rust, or poor handling [11]. The diameter, length, and position of the fragment within the root canal can all influence its nonsurgical or surgical removal. Periapical surgery in the mandibular premolar and molar areas presents certain technical difficulties regarding the proximity of the apices to the mandibular canal [12]. Further, it is essential to minimize the surgical invasion to protect the surrounding healthy tissue during foreign body removal from the jaw [8]. Therefore, as in the present case, it was necessary to carefully remove the instrument that migrated into the jawbone. Multiple methods of localizing and removing broken dental instruments have been described, including plain film radiography, fluoroscopy, image intensifiers, reference markers, and even magnets [13–15].

In this case, an intraoperative navigation system was used to locate the broken dental fragment and aid in its removal. Navigation was initially developed for stereotactic interventions in neurosurgery but has been recently introduced to other specialties. The use of navigation systems to retrieve foreign bodies during craniomaxillofacial surgery has been previously reported [16, 17].

Image-guided systems can improve preoperative planning and provide high-degree intraoperative accuracy and precision. However, the navigational accuracy is limited by the system used, the method of obtaining the imaging data, and syncing the imaging data with the patient’s actual position during the procedure. Some limitations of the currently available image-guided systems should be considered because most were originally developed for neurosurgical purposes [18]. Therefore, care must be ensured when performing oral-maxillofacial surgery, particularly in the mandible region, as it is not approved for use in this region because of the constant movement of this area. However, if the mandible were to be held in an identical position during image acquisition and the surgical procedure, then it can be assumed that all structures within the image would be fixed in an identical position, thereby enabling the use of the navigation system in its intended fashion. It was difficult to use navigation surgery in the mandible as the mobile nature of the mandible complicates its synchronization with the preoperative imaging data during surgery.

Furthermore, there are currently three possible solutions for the application of navigation in the mandible. The first approach is to mount a dynamic reference frame to the mandible that enables continuous tracking of the mandibular movement and its position during the surgery [19]. This method enables the direct tracking of the mandible via a tooth-mounted sensor frame and tooth-supported fiducial markers useful for mandibular navigation. Using this approach, the mandible is allowed to freely move during the surgery. However, the fixation of the reference requires a special procedure and is more time-consuming and complicated. In addition, the reference frame may influence the operation, possibly losing its position. The second method is an intermaxillary fixation. By maintaining an immobile intercuspal position, mandibular synchronization can be intraoperatively ensured [20]. However, this approach considerably limits access to the surgical site and is not feasible for transoral surgery. The third strategy is to position the mandible in a reproducible posture or a defined position against the maxilla, using an occlusion splint. Although artificial fixation of the mandible via a template appears to introduce no additional error, this strategy is sensitive to the relative movement of the mandible, which in turn reduces the accuracy of the navigation system [21].

In the present case, a navigation system for the mandibular lesion was further beneficial in determining the accurate location of the object and provided us, the oral-maxillofacial surgeons, with intraoperative guidance for the safe and reliable use of an individual occlusion splint. This splint, which could be easily made from a simple impression, used each tooth of the maxilla and mandible as a fixed source. Therefore, it is possible to reproduce the mandibular position for various mandibular movements. This method also has the advantages of improving surgical and oromandibular anatomical accuracy, manifesting precise individual dental root positions, minimizing surgical invasiveness, and reducing operation time using an easily constructed splint.

## Conclusions

The use of a surgical navigation system together with an interocclusal splint enabled the retrieval of a broken dental instrument in a safe and minimally invasive manner without damaging nearby vital structures.

## Abbreviations

CT: computed tomography
